# Focused Training for Humanitarian Responders in Regional Anesthesia Techniques for a Planned Randomized Controlled Trial in a Disaster Setting

**DOI:** 10.1371/currents.dis.e75f9f9d977ac8adededb381e3948a04

**Published:** 2016-11-16

**Authors:** Adam R. Aluisio, Carrei Teicher, Tess Wiskel, Allysia Guy, Adam Levine

**Affiliations:** Warren Alpert Medical School, Brown University, Providence, Rhode Island, USA; Epicentre, Paris, France; Médecins Sans Frontières USA, New York, New York, USA; Warren Alpert Medical School, Brown University, Providence, Rhode Island, USA; Lincoln Medical and Mental Health Center, Bronx, New York, USA; Warren Alpert Medical School, Brown University, Providence, Rhode Island, USA

## Abstract

Background:****Lower extremity trauma during earthquakes accounts for the largest burden of geophysical disaster-related injuries. Insufficient pain management is common in disaster settings, and regional anesthesia (RA) has the potential to reduce pain in injured patients beyond current standards. To date, no prospective research has evaluated the use of RA in a disaster setting. This cross-sectional study assesses knowledge translation and skill acquisition outcomes for lower extremity RA performed with and without ultrasound guidance among a cohort of Médecins Sans Frontières (MSF) volunteers who will function as proceduralists in a planned randomized controlled trial evaluating the efficacy of RA for pain management in an earthquake setting.

Methods:****Generalist humanitarian healthcare responders, including both physicians and nurses, were trained in ultrasound guided femoral nerve block (USGFNB) and landmark guided fascia iliaca compartment block (LGFICB) techniques using didactic sessions and interactive simulations during a one-day focused course. Outcome measures evaluated interval knowledge attainment and technical proficiency in performing the RA procedures. Knowledge attainment was assessed via pre- and post-test evaluations and procedural proficiency was evaluated through monitored simulations, with performance of critical actions graded by two independent observers.

Results:****Twelve humanitarian response providers were enrolled and completed the trainings and assessments. Knowledge scores significantly increased from a mean pre-test score of 79% to post-test score of 88% (p<0.001). In practical evaluation of the LGFICB, participants correctly performed a median of 15.0 (Interquartile Range (IQR) 14.0-16.0) out of 16 critical actions. For the USGFNB, the median score was also 15.0 (IQR 14.0-16.0) out of 16 critical actions. Inter-rater reliability for completion of critical actions was excellent, with inter-rater agreement of 83.3% and 91.7% for the LGFICB and USGFNB evaluations, respectively.

Discussion:****Prior to conducting a trial of RA in a disaster setting, providers need to gain understanding and skills necessary to perform the interventions. This evaluation demonstrated attainment of high knowledge and technical skill scores in both physicians and nurses after a brief training in regional anesthesia techniques. This study demonstrates the feasibility of rapidly training generalist humanitarian responders to provide both LGFICB and USGFNB during humanitarian emergencies.

## Background

Between 1994 and 2013, approximately 7000 natural disasters were reported, affecting more than 200 million people and accounting for over one million deaths globally. Among geophysical disasters, earthquakes result in high mortality rates and account for the largest burden of injuries^1^^,^ 2. Research from multiple settings demonstrates that earthquake-associated trauma most commonly causes injuries to the lower extremities^3^^,^ 4 ^,^ 5 ^,^ 6. Inadequate pain management during natural disasters, where resources are often constrained, is common and can result in both short-term and long-term physiologic and psychological sequela among an already high-risk patient population^7^^,^ 8 ^,^ 9 ^,^ 10.

Prior studies have demonstrated that regional anesthesia (RA) is a rapid and safe method for reducing pain caused by lower extremity trauma, and as such may have a role in improving pain management during the acute response phase of a major earthquake^11^^,^ 12 ^,^ 13 ^,^ 14 ^,^ 15. However, most studies on RA have been conducted in high-resource settings by specially trained proceduralists and have only enrolled patients with simple hip or femur fractures, which may not be generalizable to the more complex injury pattern typical of earthquake-related trauma^16^^,^ 17 ^,^ 18. There are anecdotal reports of the use of both landmark guided (LG) and US-guided (USG) RA for treatment of earthquake-related injuries, suggesting feasibility in the use of these modalities in disaster settings^9^^,^ 19 ^,^ 20. However, there have been no high-quality studies conducted evaluating the effectiveness, safety, or acceptability of RA in the aftermath of a major earthquake.

The Regional Anesthesia for Painful Injuries after Disasters (RAPID) study is randomized controlled trial (RCT) that will be carried out in the immediate aftermath of a major earthquake to determine whether RA provided by generalist humanitarian medical responders, either with or without ultrasound-guidance, can improve pain treatment for lower limb injuries, above current standards of care^21^. The RAPID study will be implemented by Médecins Sans Frontières (MSF) personnel deployed in response to an earthquake in a low- or middle-income countries (LMIC) setting^21^^,^ 22 ^,^ 23. Limited prior research on training non-specialist providers in RA in pre-hospital and emergency department settings has demonstrated successful skill attainment^24^^,^ 25 ^,^ 26 ^,^ 27. Although this evidence supports the concept of generalist humanitarian providers being capable of performing RA, no data exists pertaining specifically to this population, who provide care in unique practice environments. This study assesses knowledge translation and skill acquisition for simulated lower extremity RA performed both with and without ultrasound guidance by a cohort of MSF association members and describes the focused training methodology used.

## Methods

*Ethics*

The RAPID study has received ethical approval from the Médecins Sans Frontières Ethical Review Board (Reference number: 1524) and has been preregistered at ClinicalTrials.gov (number: NCT02698228)^21^. All training participants provided written informed consent for study activities which were conducted in accordance with the Declaration of Helsinki.

*Study Design Setting and Population*

This cross-sectional study was designed to evaluate the efficacy of a focused training in regional anesthesia for lower extremity injuries provided to MSF volunteers who will serve as research proceduralists in a future RCT of RA for pain management in earthquake victims^21^. Study activities were carried out during a one-day training in June of 2015 at the MSF-USA office (New York City, United States).

The study population was comprised of physician and nurse responders who had previously been deployed to humanitarian emergencies and were members of the MSF-USA association. Participants were made aware of the RAPID study and training prior to the meeting via digital correspondence with study investigators and were solicited to volunteer for participation.

*Participant Training and Assessment*

Training activities were designed to provide understanding of RA principles and competence in performing landmark guided fascia iliaca compartment blocks (LGFICB) and ultrasound-guided femoral nerve blocks (USGFNB). Study throughput is outlined in Figure 1. Participants completed a baseline knowledge assessment prior to the provision of training activities. The pre-assessment was comprised of fifteen multiple-choice questions that covered topics pertaining to techniques for general RA and specifically to LGFICB and USGFNB. Training and assessment materials were derived from joint committee recommendations for education and training in ultrasound-guided interventional pain procedures from American, European and Australasian international pain and anesthesia societies^28^. Participants were randomly allocated to take one of two versions of the assessment exam (test A or test B), with each exam having fifteen questions which were unique in both query stems and possible responses but which covered the same content matter and key concepts.

Following the initial assessment, participants took part in a three-hour interactive didactic session lead by study investigators using a semi-structured discussion platform that allowed for open-form content exchange between all participants. The educational information focused on RA principles and specific methodologies in performing LGFICB and USGFNB^28^. Additional information relating to the RAPID study protocols was also provided. Given the evidence supporting the use of simulation in teaching invasive procedures^29^, participants took part in three simulation-based training sessions subsequent to the didactic session. These three sessions focused on performance of USGFNB, LGFICB and general ultrasound-guided needle injection. The duration of each practical session was 45 minutes, with a 3:1 participant to instructor ratio in each session. All instructors were board certified physicians in either anesthesia or emergency medicine or senior emergency medicine residents with experience in RA techniques. The LGFICB sessions were taught using a live model simulation with a focus on surface anatomy and technical aspects specific to the procedure. For the USGFNB sessions, a femoral regional anesthesia ultrasound model (CAE Healthcare™, model number: BPF1400) was used, focusing on technical aspects of the procedure specific to perineural anesthetic delivery. For the ultrasound-guided needle injection training, regional anesthesia ultrasound training block models (CAE Healthcare™, model number: BPNP150) were used and participants were able to perform multiple injections and practice needle control and delivery under ultrasound guidance with instructor support. All ultrasound trainings and evaluations were performed using Sonosite Edge™ and Terason uSmart™ 3200T ultrasound machines with high-frequency 6-13MHz linear transducers.

After completion of training activities, each participant was assessed through two observed simulation exams for performance of the LGFICB and the USGFNB. In each exam, participants were asked to perform the RA techniques using standard clinical equipment and a femoral regional anesthesia ultrasound model (CAE Healthcare™, model number: BPF1400). During the simulation exams, two independent reviewers assessed each participant’s technical competency using a preformed critical actions checklist with an objective scoring system. Assessed parameters evaluated appropriate positioning; identification of anatomic and ultrasonographic landmarks; use of sterile technique; provision of local superficial anesthetic; needle delivery; anesthetic injection; and proper procedural monitoring for possible adverse events.

A post-training, fifteen question multiple-choice exam was performed to assess interval knowledge attainment. In the post-training assessment, participants were crossed-over to complete the alternative exam to the one they took at baseline (i.e., those initially allocated to test A completed test B and those which started with test B completed test A).

*Data Collection Outcome Measures*

Baseline data on participants was collected using a structured questionnaire. Information on demographics, healthcare training, humanitarian response activities, ultrasound and RA experience was gathered. Knowledge assessment exams were scored with a single best answer for each question and participants were assigned a percent correct for both the pre-test and post-test. During observed simulation exams for the RA techniques, each rater provided a critical action completion score ranging from zero to sixteen based on eight assessment parameters. Accrued data was de-identified and entered into a password-protected database that was accessible only by study personnel.

The primary outcome measures were the change in knowledge attainment based on mean pre- and post-test scores on the standardized exams and demonstration of technical competency in performing the LGFICB and USGFNB RA techniques. Technical competency was assessed based on adequate performance of critical actions during simulation assessments. Secondary outcome measures included the inter-rater reliability for the standardized critical action checklist and separate subgroup analyses of physician and nurses.

**Assessment and Training Throughput:**
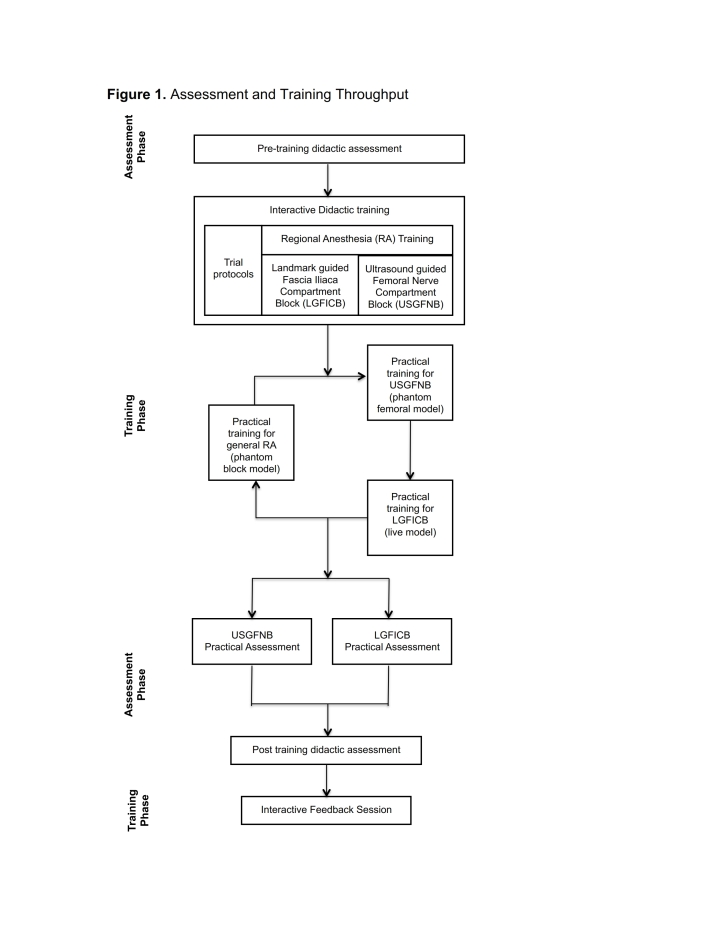

*Statistical Analysis*

Statistical analyses were performed using STATA 13.0 (StataCorp LP, College Station, USA). Characteristics of the cohort were evaluated using descriptive methods. Categorical variables were explored using frequencies with percentages, and continuous variables were analyzed using medians with corresponding interquartile ranges (IQR) or means with associated 95% confidence intervals (95% CI). Given the small sample size knowledge attainment data was analyzed using Shapiro-Wilks tests and frequency distributions to evaluate for normality. The results were found to satisfy criteria for normality and as such, one sample paired t-tests were used for the primary outcome of change in mean knowledge scores. A p value less than 0.05 was considered significant in primary outcome assessments.

Median scores with IQR for each rater were calculated for the LGFICB and USGFNB simulation assessments. Inter-rater reliability (IRR) was evaluated across all critical assessment variables based on rater agreement derived using Cohen’s kappa calculation. Kappa values were interpreted according to previously utilized criteria with agreement classified as greater than 0.80 as representing excellent IRR^30^^,^ 31. Stemming from inherent difference in healthcare training and practice roles, outcomes for the cohort were stratified and analyzed based on participants’ professional training as a nurse or physician. Subgroups were compared using independent sample t tests or Mann-Whitney U tests for normally and non-normally distributed variables respectively. To account for multiple testing, a Bonferroni correction was utilized and a significance level of less than 0.008 was used for stratified analyses^32^.

## Results

Twelve MSF association members were enrolled and all participants completed the full study training and assessment procedures. The median age of the cohort was 41 years and the majority of participants were female. Half of participants reported their primary healthcare training as nurse and half as physician. The median years of healthcare practice and humanitarian response experience were eleven and five years respectively. Half of the cohort had prior experience with ultrasound use, though only a minority reported previous clinical application of RA techniques ( Table 1).

**Table 1. Cohort Characteristics:** * MSF abbreviates Médecins San Frontières

Characteristics	n (%) / Median (IQR)
Age (years)	41 (35, 48)
Sex	
Male	2 (16.7%)
Female	10 (83.3%)
Primary healthcare training	
Nures	6 (50.0%)
Medical Doctor	6 (50.0%)
Duration providing healthcare (years)	11 (10, 21)
Duration as MSF^*^ response personnel (years)	5 (3, 9)
Number of response missions performed with MSF^*^	4 (4, 7)
Prior clinical use of ultrasound	
No	6 (50.0%)
Yes	6 (50.0%)
Prior clinical use of regional anesthesia	
No	10 (83.3%)
Yes	2 (16.7%)

The primary study outcomes are summarized in Table 2. Training activities resulted in a significant increase in mean knowledge assessment scores of 9.2% (p<0.001). The mean pre-training and post-training scores were 79.2% and 88.4%, respectively. Median values for achieved critical actions during simulation assessments were all greater than or equal to 15 as scored by the independent raters. Overall an excellent degree of IRR was observed between rater evaluations for the simulated RA assessments. The IRR agreement for technical assessments of the LGFICB and USGFNB were 83.3% and 91.7% respectively ( Table 2).

**Table 2. Didactic Knowledge and Procedural Skills Outcomes:** ^*^ Values represent the percent mean score with associated 95% confidence intervals (95% CI),^ #^ LGFICB abbreviates landmark guided fascia iliaca block and USGFNB abbreviates ultrasound guided femoral nerve block, ^†^ Reported as median score with associated interquartile range (IQR) out of possible score of sixteen required critical actions

	Pre-training	Post-training	Percent Change	p
Didactic Knowledge assessment score^*^	79.2 (73.9-84.4)	88.4 (82.3-94.2)	9.2%	<0.001
	Rater 1^†^	Rater 2^†^	Kappa	-
LGFICB assessment^#^	15.0 (14.0, 16.0)	15.0 (15.0, 16.0)	0.83	-
USGFNB assessment^#^	15.0 (14.0, 16.0)	15.5 (14.5, 16.0)	0.92	-

Table 3 demonstrates stratified outcome assessments based on participants’ healthcare training. There were no significant differences identified in knowledge attainment or technical skill performance between the physician and nurse subgroups.

**Table 3. Outcome Comparison Based on Primary Healthcare Training:** ^*^ Values represent the percent mean score with associated 95% confidence intervals (95% CI), ^#^ LGFICB abbreviates landmark guided fascia iliaca block and USGFNB abbreviates ultrasound guided femoral nerve block, ^†^ Reported as median score with associated interquartile range (IQR), maximum possible score out of sixteen required critical actions

Assessment Type	Nurse	Physician	p
Pre-training didactic exam^*^	78.7 (74.3-83.1)	79.6 (67.6-91.7)	0.86
Post training didactic exam^*^	91.7 (85.6-97.8)	85.2 (73.7-96.1)	0.23
LGFICB assessment^#^			
Rater 1^**†**^	14.5 (14.0, 15.0)	14.0 (14.0, 16.0)	0.18
Rater 2^**†**^	15.0 (14.0, 16.0)	15.0 (15.0, 16.0)	0.06
USGFNB assessment#			
Rater 1†	15.5 (15.0, 16.0)	14.0 (14.5, 16.0)	0.35
Rater 2†	15.5 (14.0, 16.0)	15.5 (15.0, 16.0)	0.80

## Discussion

This study assessed knowledge translation and skill acquisition outcomes for simulated lower extremity RA performed both with and without ultrasound guidance among a small cohort of MSF volunteers who will function as proceduralists in a planned randomized controlled trial that will assess the efficacy of RA for pain management in the acute phase of a major earthquake^21^. The results demonstrate high knowledge and technical skill acquisition with good agreement between independent raters. These findings illustrate success of the focused training methods used in this population of response providers in gaining proficiency in both LGFICB and USGFNB regional anesthesia.

In the acute response phase to a natural disaster, where resources are often constrained, oligoanalgesia is common^7^^,^ 8 ^,^ 9. Standard pain management in disaster settings for severe lower limb injuries focuses on the use of parenteral narcotic medications^33^^,^ 34. However, such medications are frequently unavailable creating preexistent barriers to analgesic treatment in low-income disaster settings^35^^,^ 36. Additionally, even when narcotic medications are accessible patients with trauma often have tenuous hemodynamic states, making administration of narcotics clinically unfavorable, especially in situations where continuous cardiorespiratory monitoring and personnel are limited. Subsequently, insufficient pain management can potentiate both short-term and long-term sequela, including immunosuppression, thrombotic complications, posttraumatic stress disorders, and chronic pain syndromes, further increasing the morbidity associated with the index injuries^8^^,^ 10. Anecdotal reports and recent review articles have suggested that regional anesthesia has the potential to improve pain control in disaster settings^9^^,^ 10 ^,^ 19 ^,^ 20 ^,^ 37. However, the published literature on the use of RA in disasters is limited, and to date no randomized controlled trial has evaluated the efficacy, safety and acceptability of nerve blocks in a disaster context. The planned RAPID study will address these specific gaps in the research^21^. A key step in executing the trial is appropriately training personnel to carry out the procedures and protocols. This study provides evidence of success in personnel training for a small cohort of generalist humanitarian responders to perform RA.

Standardized assessment and training in research procedures is important in reducing error and deriving valid trial results^38^. The present study used rigorous methodologies that integrated both interactive didactic sessions and multimodal simulation to train providers in focused RA. The employed training principles are similar to prior documented works in procedural skills and RA training that have utilized systematic assessments of technical skills^39^^,^ 40. Additionally, the assessment methods for technical competency in evaluating procedural abilities were checklist-based, which have been shown to result in more thorough and objective assessments of component skills in multi-step medical procedures^41^^,^ 42. Furthermore, the assessment outcomes for procedural performance, evaluated by independent raters, overall demonstrated excellent agreement, supporting validity in the results^31^^,^ 43. These findings, in conjunction with the significant improvement in interval didactic assessment scores in both the full cohort and individual professional sub-groups, support the efficacy of the training methodology.

Although prior studies have assessed the performance of femoral never blockades after brief trainings in emergency department settings among physician and nurse trainees this study is the first to evaluate formal training of generalist humanitarian response providers in RA methods^24^^,^ 25 ^,^ 44 ^,^ 45. In addition, a prehospital study of pain management for lower extremity injuries performed by emergency medical service providers found that approximately 89% of patients had effective analgesic treatment with provision of prehospital RA^26^. This prehospital efficacy was replicated in a cohort of paramedic proceduralists employing fascia iliaca compartment blocks in a controlled trial among patients with femur fractures^27^. The congruent findings in successful RA techniques between the prehospital and hospital based studies and the present work supports validity in the findings and bolsters the feasibility of pre-training generalist providers to perform RA in an acute disaster setting.

This work must be interpreted in the context of certain limitations. The presented results illustrate short-term acquisition of knowledge and skills among the MSF volunteers trained, but do not allow for assessment of application in actual disaster situations, nor for evaluation of skill retention over time. It is likely that similar to most technical skill sets, the study proceduralists will require refresher training prior to execution of the RAPID study, which has been built into the trial design. Although the population of humanitarian responders was drawn from a large pool of MSF volunteer personnel, the cohort was relatively small and not randomly selected, and as such the generalizability of these outcomes to the greater population of disaster healthcare personnel is not certain. During completion of the RAPID study this will be partially addressed, as there will be further onsite training for locally recruited healthcare providers, who will also serve as proceduralists during the later study phases. These later phase providers will be trained and assessed onsite using the same methods as described in this report.

Prior to conducting a trial of RA in a disaster setting, providers need to gain the knowledge and skills necessary to perform the interventions. This study demonstrated high knowledge and technical skills scores with excellent inter-rater agreement between independent evaluators, illustrating the success of the focused training for generalist humanitarian response providers in the selected RA techniques. This work, in conjunction with the planned RAPID study, will contribute to the development of an enhanced evidence base to guide future care and improve health outcomes among high-risk patients injured in the settings of humanitarian emergencies.

## Data Availability Statement

The data used for this analysis can be accessed via: https://figshare.com/s/1825267cbf21863b6cf4.

## Competing Interests

All authors declare that they have no competing interests to disclose.

## Corresponding Author

Adam R. Aluisio, MD, MSc

Warren Alpert School of Medicine, Brown University, Department of Emergency Medicine

55 Claverick Street, Room 274

Providence, Rhode Island 02912, USA

Email: adam.aluisio@gmail.com

## Appendices: Abbreviations

CRED: Centre for Research on the Epidemiology of Disasters

FICB: Fascia iliaca compartment block

FNB: Femoral nerve block

IQR: Interquartile Range

IRR: Inter-rater reliability

LG: Landmark guided

LGFICB: Landmark guided fascia iliaca compartment block

LMIC: low- and middle-income countries

MoH: Ministry of Health

MSF: Médecins Sans Frontières

RA: Regional anesthesia

RCT: Randomized Controlled Trial

SAE: Serious Adverse Events

SD: Standard Deviation

US: Ultrasound

USG: Ultrasound guided

USGFNB: Ultrasound guided femoral nerve block

WHO: World Health Organization
