# Correction: Focused Training for Humanitarian Responders in Regional Anesthesia Techniques for a Planned Randomized Controlled Trial in a Disaster Setting

**DOI:** 10.1371/currents.dis.14d4a293a1f174e3fd95c1caa63bc2bf

**Published:** 2017-02-06

**Authors:** PLOS Currents

## Correction

The second author's name is spelled incorrectly. The correct name is: Carrie Teicher. The correct citation is: Aluisio AR, Teicher C, Wiskel T, Guy A, Levine A. Focused Training for Humanitarian Responders in Regional Anesthesia Techniques for a Planned Randomized Controlled Trial in a Disaster Setting. PLOS Currents Disasters. 2016 Nov 16 . Edition 1. doi: 10.1371/currents.dis.e75f9f9d977ac8adededb381e3948a04.
